# Myocarditis masquerading as acute coronary syndrome: diagnostic role of cardiac MRI

**DOI:** 10.1186/1532-429X-18-S1-P174

**Published:** 2016-01-27

**Authors:** Bethany Wisotzkey, Brian Soriano, Erin Albers, Mark R Ferguson, Sujatha Buddhe

**Affiliations:** 1 grid.240741.40000000090264165Pediatric cardiology, Seattle Childrens's Hospital, Seattle, WA USA; 2 grid.240741.40000000090264165Pediatric radiology, Seattle Children's hospital, Seattle, WA USA

## Background

Myocarditis presenting as isolated acute chest pain with elevated troponins but normal systolic function by echocardiogram is rare in previously healthy children. Diagnosis is challenging in this situation with significant drawbacks even for the gold standard endomyocardial biopsy. Our study aim is to evaluate the diagnostic role of cardiac MRI in comparison with echocardiography in these patients.

## Methods

All children who underwent cardiac MRI (CMR) for evaluation of sudden acute chest pain with elevated troponins from April 2010 to May 2015 at our institution were included in the study and their findings were compared to age-matched controls with normal CMR. Echocardiographic parameters of left ventricular (LV) function included: subjective assessment, fractional shortening (FS%), ejection fraction (EF%) and speckle-tracking-derived strain from vector velocity imaging (TomTec Imaging Systems GmBH, Munich, Germany). CMR parameters included: LVEF%, T2 imaging, late gadolinium enhancement (LGE), and tissue-tracking-derived global peak longitudinal, radial and circumferential strain.

## Results

Group I included 9 subjects compared with 10 controls in group II. There were 8 males (89%) in group I vs. 7 males (70%) in group II (p=0.6). All 9 subjects had LGE consistent with myocarditis: 8 in left ventricle and 1 in right ventricle. Troponin level ranged from 2.5 - 22.9 ng/ml and did not correlate with the extent of myocardial LGE involvement. EKG was concerning for ST segment elevation in 5 and abnormal Q waves in 1 subject. Qualitative echocardiographic function was normal in all children in both groups, while 2 subjects had decreased function by CMR. Mean echo FS% was not different between groups (36 ± 5 % vs. 35 ± 4 %) while MRI LVEF% had a trend to be lower in subjects (54 ± 5 % vs. 59 ± 4 %; p= 0.06). MRI derived strain parameters were lower in subjects compared to controls for global peak longitudinal (-12.8 ± 2.8 % vs. -17.1 ± 1.5 %; p = 0.05), circumferential (-12.3 ± 3.8 % vs. -15.8 ± 1.2 %; p = 0.03) and radial strains (13.6 ± 3.7 % vs. 17.2 ± 3.2 %; p = 0.04) respectively. Echocardiography derived strain parameters also were lower in subjects compared to controls for global peak longitudinal (-16.3 ± 3.5 % vs. -20.8 ± 2.2 %; p < 0.01), circumferential (-16.5 ± 2.7 % vs. -19.8 ± 1.9 %; p< 0.01) and radial strains (17.3 ± 6.5 % vs. 24.8 ± 6.3 %; p = 0.02) respectively. Follow-up MRI in 4 patients had persistent LGE findings at 1.0 - 2.1 years.

## Conclusions

In previously asymptomatic children, myocarditis can present with symptoms of acute chest pain suspicious for coronary ischemia. CMR is a non-invasive, radiation-free diagnostic test of immense diagnostic utility in these situations where clinical picture, EKG and echocardiogram are insufficient to make the diagnosis. In addition to LGE, strain imaging may also aid in diagnosis. Long term studies are needed to assess prognostic significance of these CMR findings.

